# Supplementary figures and images for: Expression of the cancer-associated DNA polymerase ε P286R in fission yeast leads to translesion synthesis polymerase dependent hypermutation and defective DNA replication
Source: PLoS Genet. 2021 Jul 6;17(7):e1009526. doi: 10.1371/journal.pgen.1009526 (PMC8284607; doi:10.1371/journal.pgen.1009526)

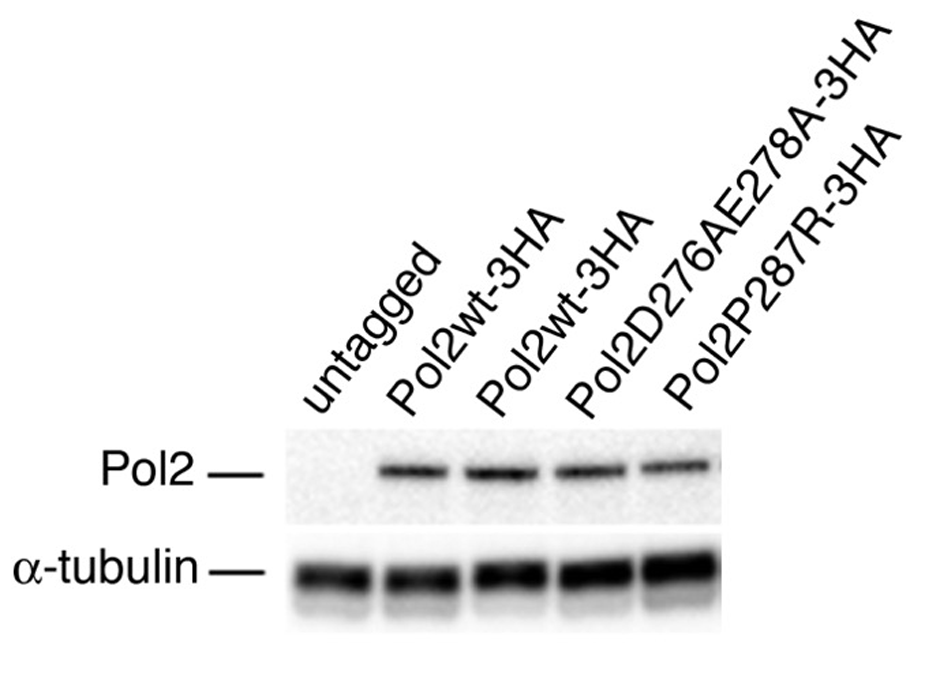

Supplement: S1 Fig — Epitope-tagged polymerases were detected using anti-HA monoclonal antibody. α-tubulin is shown as a loading control. (TIF) [file pgen.1009526.s001.tif]

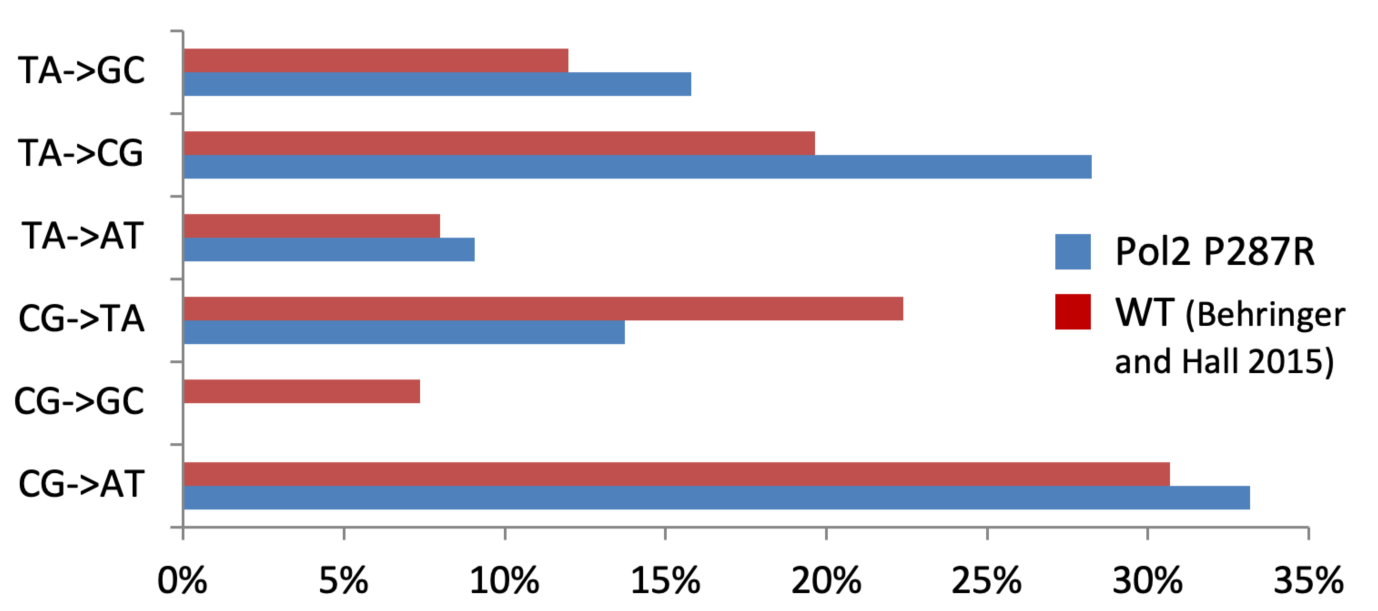

Supplement: S2 Fig — (TIF) [file pgen.1009526.s002.tif]

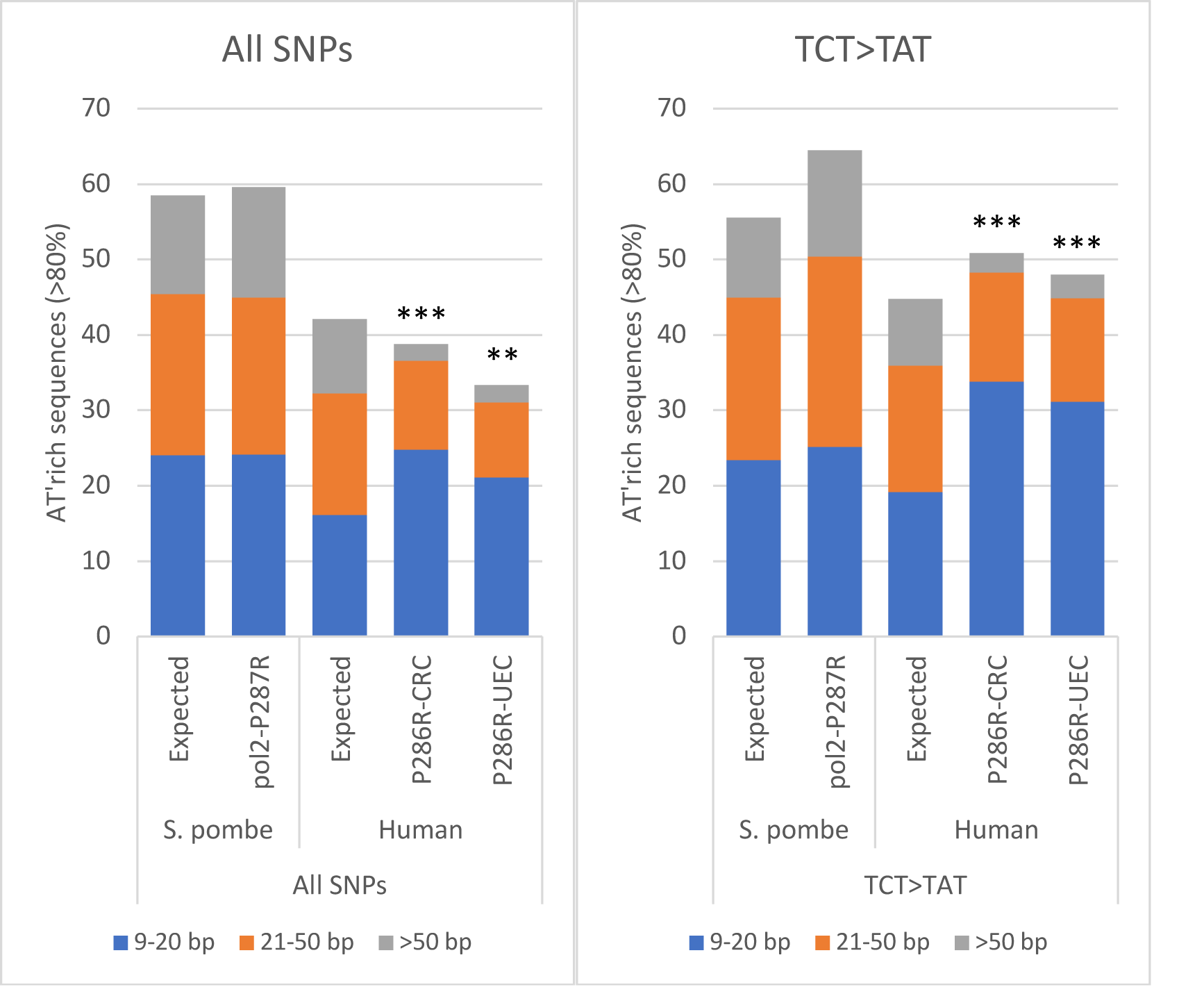

Supplement: S3 Fig — Corresponding numerical data is shown in S6 Table. Differences from expected frequency is significant for human 9–20 bp AT-rich sequences (*** p<0.01; ** p<0.05). (TIF) [file pgen.1009526.s003.tif]

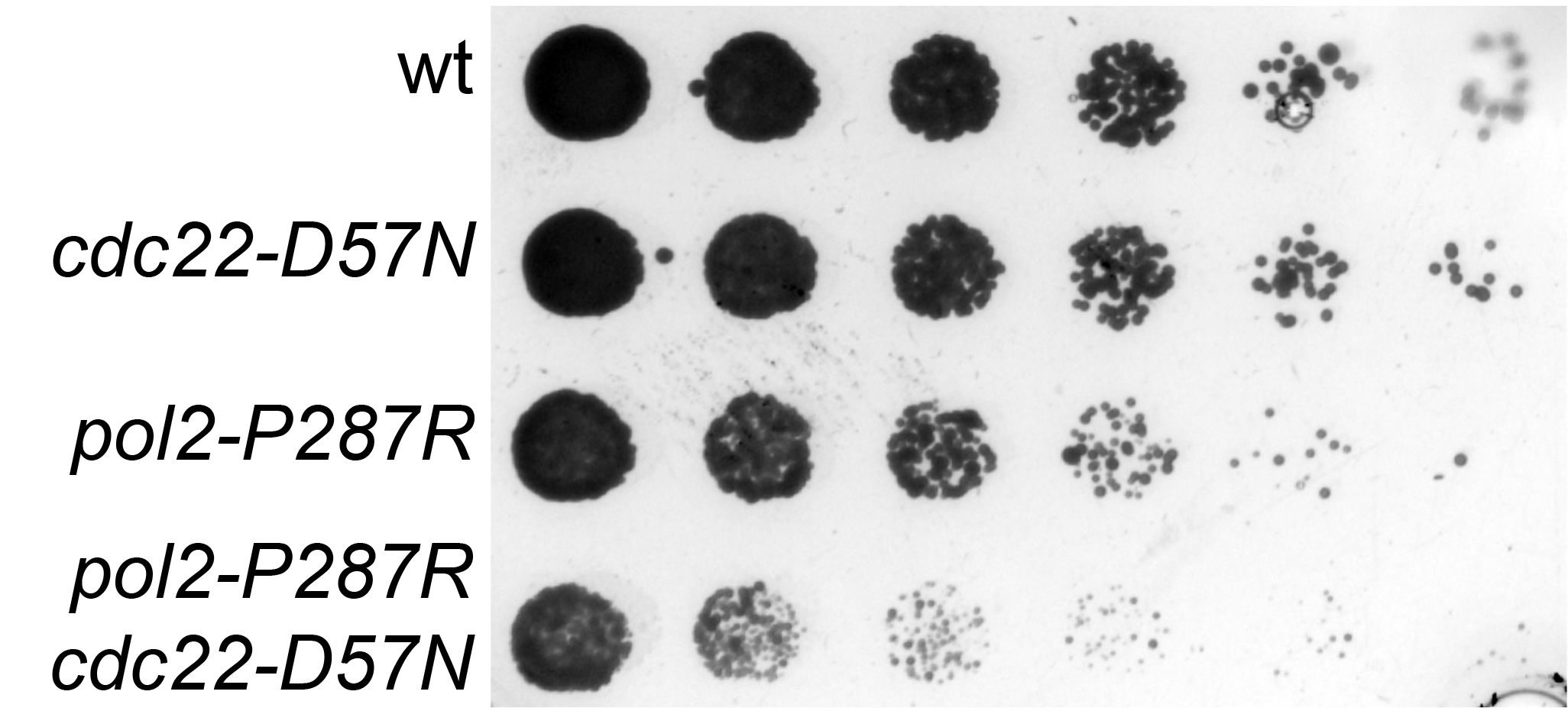

Supplement: S4 Fig — (TIF) [file pgen.1009526.s004.tif]

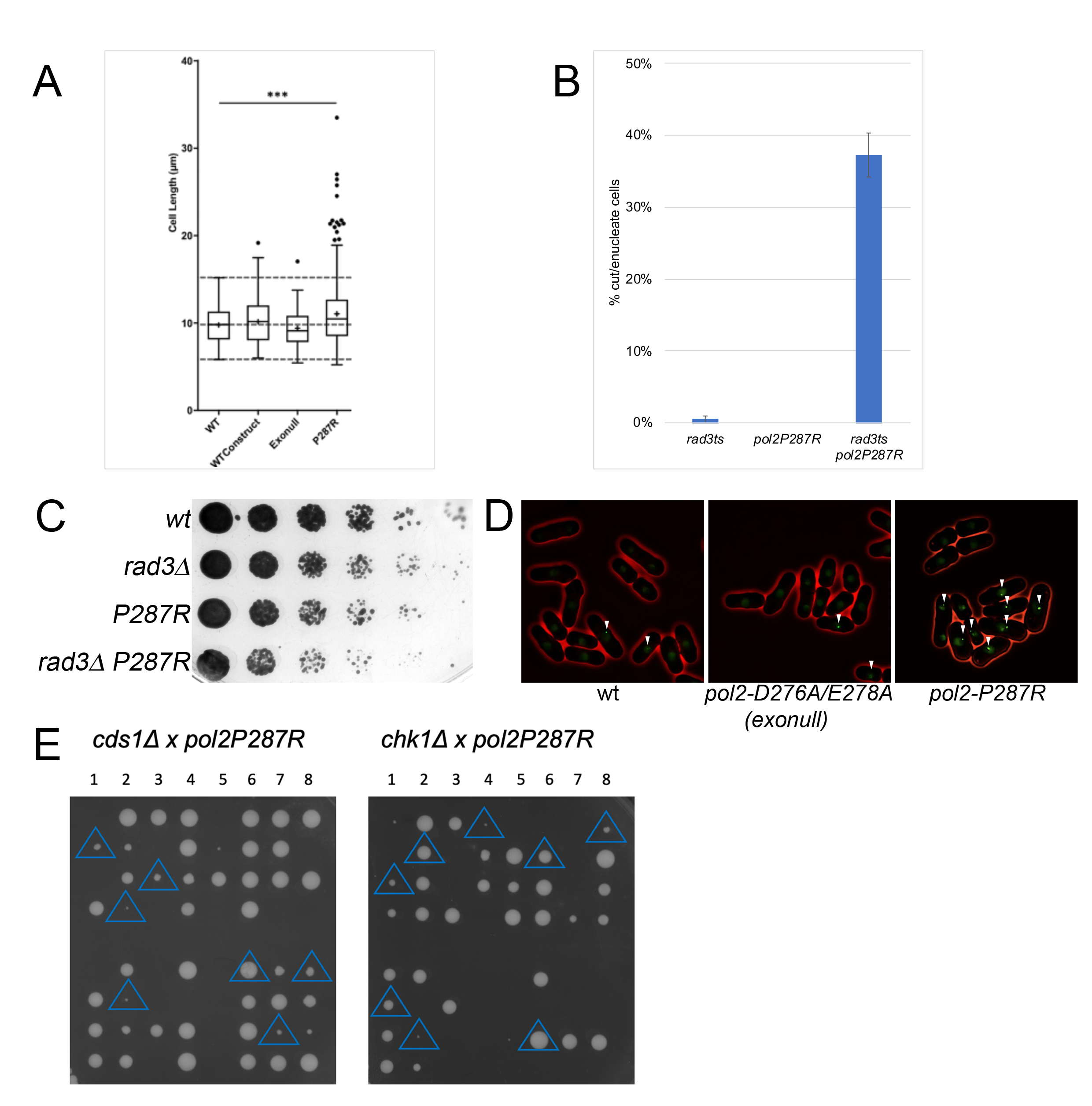

Supplement: S5 Fig — (A) Cell length measurements of S. pombe cells in log phase. 200–400 cells were measured for each strain. Statistical analysis used a Krustal-Wallis test (*** P<0.001). “Wt construct” was constructed in the same way as the other two strains except that pol2 mutations were not introduced. (B) Percentage of cut or enucleate cells in rad3ts pol2-P287R and parental strains. Log phase cells grown at 26°C were shifted to 36°C for 3 hours before examination by fluorescence microscopy. (D) Rad11-GFP (RPA) foci in live unstressed cells. Numerical data are shown in S9 Table. (E) Tetrads from cds1Δ or chk1Δ x pol2-P287R crosses. Blue triangles indicate double mutants. (TIF) [file pgen.1009526.s005.tif]

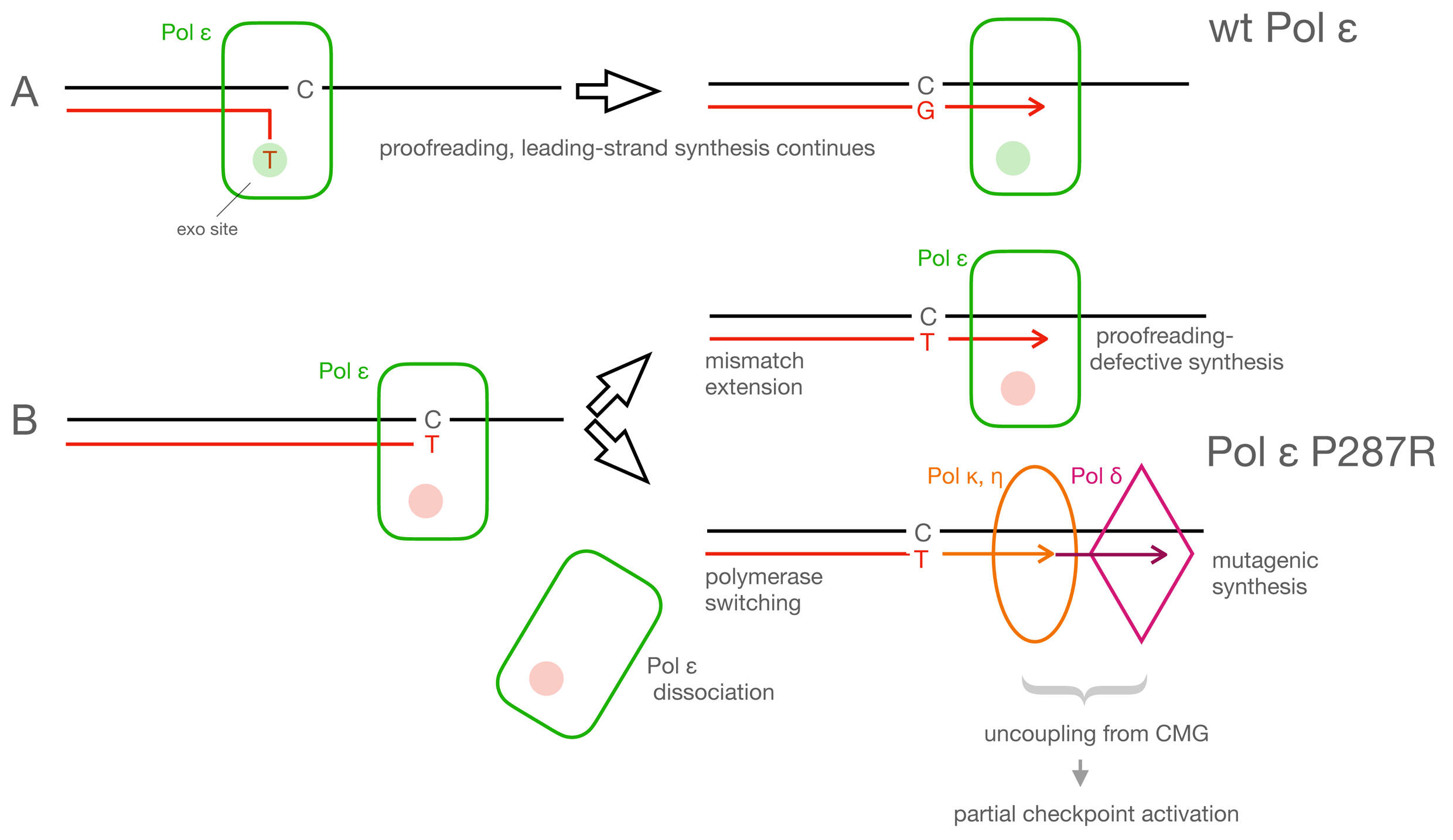

Supplement: S6 Fig — In contrast to proofreading by wt Pol ε (A), Pol2P287R can more readily extend from a mismatch (B, upper arrow). TLS polymerases (κ, η) may however facilitate switching to Pol δ (B, lower arrow). Synthesis by TLS polymerases is error prone, and DNA synthesised by Pol δ after switching may show more mutations, possibly due to defective MMR. In the absence of TLS polymerases, switching to Pol δ may be less efficient, allowing continued Pol ε synthesis. Switching or misincorporation may also result in uncoupling from the CMG and Rad3 activation. See text for further details. (TIF) [file pgen.1009526.s006.tif]
