# Supplementary material for: Expression of the cancer-associated DNA polymerase ε P286R in fission yeast leads to translesion synthesis polymerase dependent hypermutation and defective DNA replication
Source: PLoS Genet. 2021 Jul 6;17(7):e1009526. doi: 10.1371/journal.pgen.1009526 (PMC8284607; doi:10.1371/journal.pgen.1009526)
Supplement: S1 Table — (DOCX) [file pgen.1009526.s007.docx]

**S1 Table *S. pombe* strains used in this study.**

| Strain number | Genotype | Source |
| --- | --- | --- |
| 2470 | *h- adh1::adh1prom-TK::kanMX6 leu1-32::adh1prom-hENT1::leu1^+^ leu1-32* | [1] |
| 2526 | *h- leu1 ura4 cds1-2HA6His::ura4^+^* | MY3141 NBRP, Japan |
| 2527 | *chk1-HA::LEU2 ade6-216 leu1-32* | MY2167 NBRP, Japan |
| 2839 | *ade6-704 leu1-32 ura4-D18 rad3ts h-* | T. Carr |
| 2840 | *ade6-485 h-* | O. Fleck, used as wt control |
| 3199 | *pol2-D276A/E278A::kanMX6 ade6-485 h- (“exonull ε”)* | [2] |
| 3273 | *pol3-D386A::kanMX6 ade6-485 h- (“exonull δ”)* |  |
| 3285 | *cdc22-D57N h+* | C. Holmberg (EG1717) |
| 3302 | *pol2P287R::hphMX6 rad11:rad11-GFP::kanMX6 ade6 leu1 ura4 h+* |  |
| 3330 | *rev3Δ::kanMX6* |  |
| 3334 | *kpa1Δ::kanMX6 h-* | S Coulon (SC215) |
| 3336 | *rev1Δ::kanMX6 ade6-704 leu1-32 ura4-D18* | T. Carr (SR34) |
| 3344 | *kpa1Δ::kanMX6 pol2P287R::hphMX6 clone 1* |  |
| 3419 | *pol3P311R::kanMX6, ade6-485 h-* |  |
| 3622 | *pol2P287R::kanMX6 ade6 h-* |  |
| 3623 | *rad11:rad11-GFP::hphMX6 leu1-32 ura4-D18 h+* | P. Russell |
| 3653 | *eso1-D147N::kanMX6 ura4-D18 leu1-32* | E. Hartsuiker |
| 3693 | *pol2D276A/E278A::kanMX6 rad11:rad11-GFP::hphMX6* |  |
| 3704 | *cds1-2HA6His::ura4^+^ pol2P287R::kanMX6* |  |
| 3740 | *adh1::adh1prom-TK::kanMX6 leu1-32::adh1prom-hENT1::leu1+ pol2P287R::hphMX6* |  |
| 3741 | *adh1::adh1prom-TK::kanMX6 leu1-32::adh1prom-hENT1::leu1+ pol2P287R::hphMX6* |  |
| 3747 | *pol2P287R::hphMX6 rev1Δ::kanMX6* |  |
| 3753 | *rev3Δ::kanMX6 pol2P287R::hphMX6 h-* |  |
| 3754 | *pol2P287R::kanMX6 ade6-M210 leu1-32 ura4-D18 h-* |  |
| 3756 | *pol2P287R::kanMX6 ade6-M210 leu1-32 ura4-D18 h-* |  |
| 3767 | *pfh1::ura4^+^-nmt1(81X)pfh1^+^-GFP h-* | ySP383 [3] |
| 3770 | *pol2P287R::kanMX6* [pREP1-pfh1] | pREP1-pfh1 [4] |
| 3780 | *pfh1::ura4^+^-nmt1(81X)pfh1^+^-GFP pol2D276A/E278A::kanMX6* |  |
| 3823 | *pol2D276A/E278A::kanMX6 rad3ts ade6 leu1-32 ura4-D18 clone 1* |  |
| 3824 | *pol2D276A/E278A::kanMX6 rad3ts ade6 leu1-32 ura4- D18clone 2* |  |
| 3825 | *pol2D276A/E278A::kanMX6 rad3ts ade6 leu1-32 ura4- D18 clone 3* |  |
| 3826 | *pol2P287R::kanMX6 rad3ts ade6 leu1-32 ura4-D18 clone 1* |  |
| 3827 | *pol2P287R::kanMX6 rad3ts ade6 leu1-32 ura4-D18 clone 2* |  |
| 3828 | *pol2P287R::kanMX6 rad3ts ade6 leu1-32 ura4-D18 clone 3* |  |
| 3923 | *pol2^+^-3HA::natMX6 ade6-485* |  |
| 3924 | *pol2::pol2^+^-3HA::natMX6 ade6-485* |  |
| 3925 | *pol2D276A/E278A-3HA::natMX6 ade6-485* |  |
| 3926 | *pol2P287R-3HA::natMX6 ade6-485* |  |
| 4030 | *pfh1::ura4^+^-nmt1(81X)pfh1^+^-GFP pol2P287R::kanMX6* |  |
| 4031 | *eso1-D147N::kanMX6 pol2P287R::hphMX6* |  |
| 4033 | *cds1-2HA6His::ura4^+^ pol2D276A/E278A::hphMX6* |  |
| 4034 | *chk1-HA::LEU2 pol2P287R::kanMX6 clone 1* |  |
| 4035 | *chk1-HA::LEU2 pol2P287R::kanMX6 clone 2* |  |
| 4036 | *chk1-HA::LEU2 pol2D276A/E278A::kanMX6* |  |
| 4037 | *adh1::adh1prom-TK::kanMX6 leu1-32::adh1prom-hENT1::leu1+ pol2P287R::hphMX6* |  |
| 4038 | *pol2P287R::kanMX6 cdc22-D57N* |  |

1. Hua H, Kearsey SE. Monitoring DNA replication in fission yeast by incorporation of 5-ethynyl-2'-deoxyuridine. Nucleic Acids Res. 2011;39(9):e60. Epub 2011/02/12. doi: 10.1093/nar/gkr063. PubMed PMID: 21310713; PubMed Central PMCID: PMCPMC3089489.

2. Aoude LG, Heitzer E, Johansson P, Gartside M, Wadt K, Pritchard AL, et al. POLE mutations in families predisposed to cutaneous melanoma. Fam Cancer. 2015;14(4):621-8. Epub 2015/08/08. doi: 10.1007/s10689-015-9826-8. PubMed PMID: 26251183.

3. Pinter SF, Aubert SD, Zakian VA. The Schizosaccharomyces pombe Pfh1p DNA Helicase Is Essential for the Maintenance of Nuclear and Mitochondrial DNA. Molecular and Cellular Biology. 2008;28(21):6594-608. doi: 10.1128/MCB.00191-08.

4. Audry J, Maestroni L, Delagoutte E, Gauthier T, Nakamura TM, Gachet Y, et al. RPA prevents G-rich structure formation at lagging-strand telomeres to allow maintenance of chromosome ends. EMBO J. 2015;34(14):1942-58. Epub 2015/06/05. doi: 10.15252/embj.201490773. PubMed PMID: 26041456; PubMed Central PMCID: PMCPMC4547897.
