# Supplementary material for: Expression of the cancer-associated DNA polymerase ε P286R in fission yeast leads to translesion synthesis polymerase dependent hypermutation and defective DNA replication
Source: PLoS Genet. 2021 Jul 6;17(7):e1009526. doi: 10.1371/journal.pgen.1009526 (PMC8284607; doi:10.1371/journal.pgen.1009526)
Supplement: S2 Table — (DOCX) [file pgen.1009526.s008.docx]

**S2 Table Oligos used in this study**

| Oligo number | SEQUENCE 5’-3’ |
| --- | --- |
| 1192 | AGGGATCGCGAAAAACGCCTTTGCGACCTGCGCATTC |
| 1193 | TTCGCGATCCCTCTATTGACCCTGTAATTCAAATTGC |
| 1148 | TTTGGATCCGGGTGTGATCGATCATTGCATTATAGAG |
| 1076 | TGGCGCGCCCCATCTTTTTCACCAGGACATTTCATCAAATC |
| 1140 | GCAACTTCGCGATCCCTTACCTCTTGGATCGAGCAAAAAG |
| 1141 | GATCGCGAAGTTGCAGATGTTGTACCCAATTAAAACATCTG |
| 1075 | TTTGGATCCTTGAATGCGCAGGTCGCAAAGGCG |
