# Supplementary material for: Expression of the cancer-associated DNA polymerase ε P286R in fission yeast leads to translesion synthesis polymerase dependent hypermutation and defective DNA replication
Source: PLoS Genet. 2021 Jul 6;17(7):e1009526. doi: 10.1371/journal.pgen.1009526 (PMC8284607; doi:10.1371/journal.pgen.1009526)
Supplement: S3 Table — (DOCX) [file pgen.1009526.s009.docx]

**S3 Table Cosine coefficients between humanized *S. pombe* mutational patterns and COSMIC v3.1 SBS signatures**

| COSMIC Signatures | wild type (WT) | *pol2 P287R* |
| --- | --- | --- |
| SBS1_GRCh38 | 0.20 | 0.05 |
| SBS2_GRCh38 | 0.17 | 0.08 |
| SBS3_GRCh38 | 0.69 | 0.50 |
| SBS4_GRCh38 | 0.60 | 0.59 |
| SBS5_GRCh38 | 0.67 | 0.43 |
| SBS6_GRCh38 | 0.36 | 0.15 |
| SBS7a_GRCh38 | 0.25 | 0.13 |
| SBS7b_GRCh38 | 0.39 | 0.20 |
| SBS7c_GRCh38 | 0.15 | 0.13 |
| SBS7d_GRCh38 | 0.20 | 0.17 |
| SBS8_GRCh38 | 0.53 | 0.52 |
| SBS9_GRCh38 | 0.38 | 0.38 |
| SBS10a_GRCh38 | 0.14 | 0.60 |
| SBS10b_GRCh38 | 0.16 | 0.18 |
| SBS11_GRCh38 | 0.36 | 0.22 |
| SBS12_GRCh38 | 0.28 | 0.26 |
| SBS13_GRCh38 | 0.16 | 0.05 |
| SBS14_GRCh38 | 0.29 | 0.85 |
| SBS15_GRCh38 | 0.32 | 0.19 |
| SBS16_GRCh38 | 0.23 | 0.15 |
| SBS17a_GRCh38 | 0.11 | 0.16 |
| SBS17b_GRCh38 | 0.09 | 0.17 |
| SBS18_GRCh38 | 0.68 | 0.57 |
| SBS19_GRCh38 | 0.41 | 0.21 |
| SBS20_GRCh38 | 0.34 | 0.66 |
| SBS21_GRCh38 | 0.22 | 0.27 |
| SBS22_GRCh38 | 0.09 | 0.11 |
| SBS23_GRCh38 | 0.44 | 0.23 |
| SBS24_GRCh38 | 0.70 | 0.34 |
| SBS25_GRCh38 | 0.50 | 0.44 |
| SBS26_GRCh38 | 0.28 | 0.30 |
| SBS28_GRCh38 | 0.08 | 0.12 |
| SBS29_GRCh38 | 0.71 | 0.45 |
| SBS30_GRCh38 | 0.45 | 0.20 |
| SBS31_GRCh37 | 0.52 | 0.35 |
| SBS32_GRCh38 | 0.42 | 0.26 |
| SBS33_GRCh38 | 0.18 | 0.21 |
| SBS34_GRCh38 | 0.09 | 0.02 |
| SBS35_GRCh38 | 0.50 | 0.57 |
| SBS36_GRCh38 | 0.45 | 0.64 |
| SBS37_GRCh38 | 0.30 | 0.25 |
| SBS38_GRCh38 | 0.27 | 0.37 |
| SBS39_GRCh38 | 0.50 | 0.23 |
| SBS40_GRCh38 | 0.73 | 0.53 |
| SBS41_GRCh38 | 0.28 | 0.18 |
| SBS42_GRCh38 | 0.49 | 0.40 |
| SBS44_GRCh38 | 0.42 | 0.50 |
| SBS84_GRCh37 | 0.38 | 0.21 |
| SBS85_GRCh37 | 0.21 | 0.15 |
| SBS86_GRCh37 | 0.30 | 0.09 |
| SBS87_GRCh37 | 0.32 | 0.12 |
| SBS88_GRCh37 | 0.18 | 0.15 |
| SBS89_GRCh37 | 0.57 | 0.43 |
| SBS90_GRCh37 | 0.05 | 0.03 |
| SBS27_GRCh38 | 0.12 | 0.07 |
| SBS43_GRCh38 | 0.16 | 0.11 |
| SBS45_GRCh38 | 0.31 | 0.53 |
| SBS46_GRCh38 | 0.40 | 0.35 |
| SBS47_GRCh38 | 0.24 | 0.11 |
| SBS48_GRCh38 | 0.08 | 0.01 |
| SBS49_GRCh38 | 0.26 | 0.10 |
| SBS50_GRCh38 | 0.38 | 0.20 |
| SBS51_GRCh38 | 0.35 | 0.14 |
| SBS52_GRCh38 | 0.18 | 0.14 |
| SBS53_GRCh38 | 0.29 | 0.09 |
| SBS54_GRCh38 | 0.25 | 0.24 |
| SBS55_GRCh38 | 0.12 | 0.08 |
| SBS56_GRCh38 | 0.19 | 0.62 |
| SBS57_GRCh38 | 0.28 | 0.22 |
| SBS58_GRCh38 | 0.36 | 0.22 |
| SBS59_GRCh38 | 0.33 | 0.17 |
| SBS60_GRCh38 | 0.04 | 0.05 |
