# Supplementary material for: Expression of the cancer-associated DNA polymerase ε P286R in fission yeast leads to translesion synthesis polymerase dependent hypermutation and defective DNA replication
Source: PLoS Genet. 2021 Jul 6;17(7):e1009526. doi: 10.1371/journal.pgen.1009526 (PMC8284607; doi:10.1371/journal.pgen.1009526)
Supplement: S4 Table — (DOCX) [file pgen.1009526.s010.docx]

**S4 Table Contribution of COSMIC Single Base Substitution (SBS) signatures to *S. pombe* mutational patterns**

| **COSMIC Signature** | **pol2-P287R** | **WT** |
| --- | --- | --- |
| SBS14_GRCh38 | 26.16% | 0.00% |
| SBS20_GRCh38 | 15.59% | 0.00% |
| SBS10a_GRCh38 | 11.44% | 0.00% |
| SBS35_GRCh38 | 11.41% | 0.00% |
| SBS31_GRCh37 | 6.45% | 8.81% |
| SBS21_GRCh38 | 6.15% | 0.15% |
| SBS17b_GRCh38 | 4.81% | 0.99% |
| SBS33_GRCh38 | 3.89% | 0.95% |
| SBS17a_GRCh38 | 3.58% | 0.00% |
| SBS11_GRCh38 | 3.49% | 0.00% |
| SBS26_GRCh38 | 3.34% | 7.33% |
| SBS2_GRCh38 | 1.06% | 0.63% |
| SBS28_GRCh38 | 1.00% | 0.00% |
| SBS8_GRCh38 | 0.55% | 0.00% |
| SBS22_GRCh38 | 0.53% | 0.00% |
| SBS7d_GRCh38 | 0.51% | 0.75% |
| SBS32_GRCh38 | 0.03% | 0.00% |
| SBS1_GRCh38 | 0.00% | 0.00% |
| SBS3_GRCh38 | 0.00% | 0.00% |
| SBS4_GRCh38 | 0.00% | 0.00% |
| SBS5_GRCh38 | 0.00% | 0.00% |
| SBS6_GRCh38 | 0.00% | 1.50% |
| SBS7a_GRCh38 | 0.00% | 0.00% |
| SBS7b_GRCh38 | 0.00% | 0.00% |
| SBS7c_GRCh38 | 0.00% | 0.00% |
| SBS9_GRCh38 | 0.00% | 0.00% |
| SBS10b_GRCh38 | 0.00% | 0.00% |
| SBS12_GRCh38 | 0.00% | 0.00% |
| SBS13_GRCh38 | 0.00% | 0.00% |
| SBS15_GRCh38 | 0.00% | 0.00% |
| SBS16_GRCh38 | 0.00% | 0.00% |
| SBS18_GRCh38 | 0.00% | 15.67% |
| SBS19_GRCh38 | 0.00% | 1.45% |
| SBS23_GRCh38 | 0.00% | 2.52% |
| SBS24_GRCh38 | 0.00% | 14.63% |
| SBS25_GRCh38 | 0.00% | 0.00% |
| SBS29_GRCh38 | 0.00% | 13.45% |
| SBS30_GRCh38 | 0.00% | 7.34% |
| SBS34_GRCh38 | 0.00% | 0.17% |
| SBS36_GRCh38 | 0.00% | 0.00% |
| SBS37_GRCh38 | 0.00% | 0.00% |
| SBS38_GRCh38 | 0.00% | 0.00% |
| SBS39_GRCh38 | 0.00% | 20.41% |
| SBS40_GRCh38 | 0.00% | 0.00% |
| SBS41_GRCh38 | 0.00% | 0.00% |
| SBS42_GRCh38 | 0.00% | 0.00% |
| SBS44_GRCh38 | 0.00% | 0.00% |
| SBS84_GRCh37 | 0.00% | 0.00% |
| SBS85_GRCh37 | 0.00% | 0.00% |
| SBS86_GRCh37 | 0.00% | 0.00% |
| SBS87_GRCh37 | 0.00% | 3.25% |
| SBS88_GRCh37 | 0.00% | 0.00% |
| SBS89_GRCh37 | 0.00% | 0.00% |
| SBS90_GRCh37 | 0.00% | 0.00% |
