# Supplementary material for: Expression of the cancer-associated DNA polymerase ε P286R in fission yeast leads to translesion synthesis polymerase dependent hypermutation and defective DNA replication
Source: PLoS Genet. 2021 Jul 6;17(7):e1009526. doi: 10.1371/journal.pgen.1009526 (PMC8284607; doi:10.1371/journal.pgen.1009526)
Supplement: S5 Table — (DOCX) [file pgen.1009526.s011.docx]

**S5 Table Comparison between *S. pombe* mutational patterns and *POLE* human cancer signatures**

| **Signature** | **P286R-CRC** | **P286R-CRC Normalized** | **P286R-UEC** | **P286R-UEC Normalized** | **pol2P287R** | **pol2P287R Normalized** | **pol2P287R Humanized** | **WT** | **WT Normalized** | **WT Humanized** |
| --- | --- | --- | --- | --- | --- | --- | --- | --- | --- | --- |
| **P286R-CRC** | 1 | 0.6287 | 0.9916 | 0.6169 | 0.6879 | 0.5743 | 0.5742 | 0.3106 | 0.2591 | 0.2269 |
| **P286R-CRC Normalized** | 0.6287 | 1 | 0.6545 | 0.9950 | 0.1778 | 0.1840 | 0.1426 | 0.2522 | 0.2932 | 0.1506 |
| **P286R-UEC** | 0.9916 | 0.6545 | 1 | 0.6505 | 0.6838 | 0.5926 | 0.5897 | 0.3199 | 0.2885 | 0.2460 |
| **P286R-UEC Normalized** | 0.6169 | 0.9950 | 0.6505 | 1 | 0.1731 | 0.1895 | 0.1433 | 0.2635 | 0.3221 | 0.1670 |
| **pol2P287R** | 0.6879 | 0.1778 | 0.6838 | 0.1731 | 1 | 0.9340 | 0.9164 | 0.4685 | 0.3355 | 0.3931 |
| **pol2P287R Normalized** | 0.5743 | 0.1840 | 0.5926 | 0.1895 | 0.9340 | 1 | 0.9783 | 0.4526 | 0.4045 | 0.4584 |
| **pol2P287R Humanized** | 0.5742 | 0.1426 | 0.5897 | 0.1433 | 0.9164 | 0.9783 | 1 | 0.3876 | 0.3350 | 0.4200 |
| **WT** | 0.3106 | 0.2522 | 0.3199 | 0.2635 | 0.4685 | 0.4526 | 0.3876 | 1 | 0.8822 | 0.9114 |
| **WT Normalized** | 0.2591 | 0.2932 | 0.2885 | 0.3221 | 0.3355 | 0.4045 | 0.3350 | 0.8822 | 1 | 0.9181 |
| **WT Humanized** | 0.2269 | 0.1506 | 0.2460 | 0.1670 | 0.3931 | 0.4584 | 0.4200 | 0.9114 | 0.9181 | 1 |
