# Supplementary material for: Expression of the cancer-associated DNA polymerase ε P286R in fission yeast leads to translesion synthesis polymerase dependent hypermutation and defective DNA replication
Source: PLoS Genet. 2021 Jul 6;17(7):e1009526. doi: 10.1371/journal.pgen.1009526 (PMC8284607; doi:10.1371/journal.pgen.1009526)
Supplement: S6 Table — (DOCX) [file pgen.1009526.s012.docx]

**S6 Table Percentage of mutations in AT-rich sequences**

| **AT-rich sequences (>80%)** | **All SNPs** | | | | | | **TCT>TAT** | | | | |  |
| --- | --- | --- | --- | --- | --- | --- | --- | --- | --- | --- | --- | --- |
|  | ***S. pombe*** | | ***Human*** | | | | ***S. pombe*** | | ***Human*** | | |  |
|  | **Expected** | ***pol2-P287R*** | **Expected** | | **P286R-CRC** | **P286R-UEC** | **Expected** | ***pol2-P287R*** | **Expected** | **P286R-CRC** | **P286R-UEC** |  |
| **9-20 bp** | 24.04 | 24.14 | 16.09 | | 24.78 | 21.10 | 23.39 | 25.19 | 19.14 | 33.83 | 31.18 |  |
| **21-50 bp** | 21.33 | 20.78 | 16.12 | | 11.75 | 9.91 | 21.56 | 25.19 | 16.78 | 14.41 | 13.72 |  |
| **>50 bp** | 13.12 | 14.73 | 9.94 | | 2.25 | 2.30 | 10.58 | 14.07 | 8.90 | 2.64 | 3.12 |  |
|  |  | 0.9702 |  | | 0.0073 | 0.0108 |  | 0.2971 |  | 0.0008 | 0.0071 | **P-Value** |
|  |  |  |  | | *** | ** |  |  |  | *** | *** |  |
| The result is significant at p < .01 *** | | | |  |  |  |  |  |  |  |  |  |
| The result is significant at p < .05 ** | | | |  |  |  |  |  |  |  |  |  |
| The result is significant at p < .10 * | | | |  |  |  |  |  |  |  |  |  |
