# Supplementary material for: Expression of the cancer-associated DNA polymerase ε P286R in fission yeast leads to translesion synthesis polymerase dependent hypermutation and defective DNA replication
Source: PLoS Genet. 2021 Jul 6;17(7):e1009526. doi: 10.1371/journal.pgen.1009526 (PMC8284607; doi:10.1371/journal.pgen.1009526)
Supplement: S7 Table — (DOCX) [file pgen.1009526.s013.docx]

**S7 Table: dNTP levels (arbitrary units) of *S. pombe* strains**

|  | **dCTP** | | **dGTP** | | **dTTP** | | **dATP** | |
| --- | --- | --- | --- | --- | --- | --- | --- | --- |
| **Sample** | **Average** | **StDev** | **Average** | **StDev** | **Average** | **StDev** | **Average** | **StDev** |
| **WT** | 4042 | 2144 | 4099 | 2270 | 5200 | 1827 | 2997 | 1173 |
| **pol2-exonull** | 4581 | 2212 | 4256 | 2518 | 5440 | 3335 | 4039 | 1615 |
| **pol2-P287R** | 4010 | 3506 | 3932 | 1203 | 5157 | 2219 | 4275 | 2306 |
| **cdc22-D57N** | 22875 | 5910 | 19142 | 7429 | 30290 | 7787 | 37805 | 13988 |
| **cdc22-D57N P287R** | 15731 | 574 | 26158 | 858 | 37120 | 2881 | 71511 | 11424 |
