# Supplementary material for: Expression of the cancer-associated DNA polymerase ε P286R in fission yeast leads to translesion synthesis polymerase dependent hypermutation and defective DNA replication
Source: PLoS Genet. 2021 Jul 6;17(7):e1009526. doi: 10.1371/journal.pgen.1009526 (PMC8284607; doi:10.1371/journal.pgen.1009526)
Supplement: S8 Table — (DOCX) [file pgen.1009526.s014.docx]

**S8 Table: Quantitative impact of increased dNTP levels on *pol2-P287R* mutagenesis**

| Strain | Canavanine-resistance mutation rate relative to wt | 95% CI |
| --- | --- | --- |
| Wild-type (2840) | 1 |  |
| *pol2P287R* | 191 | 149-238 |
| *pol2P287R cdc22-D57N* | 441 | 365-522 |
