# Supplementary material for: Expression of the cancer-associated DNA polymerase ε P286R in fission yeast leads to translesion synthesis polymerase dependent hypermutation and defective DNA replication
Source: PLoS Genet. 2021 Jul 6;17(7):e1009526. doi: 10.1371/journal.pgen.1009526 (PMC8284607; doi:10.1371/journal.pgen.1009526)
Supplement: S11 Table — (DOCX) [file pgen.1009526.s017.docx]

**S11 Table: Mutation rate of the *pol2P287R* strain over-expressing Pfh1**

| Strain | Growth conditions | Mutation rate relative to wt | 95% CI |
| --- | --- | --- | --- |
| Wild-type (2299) | EMM | 1 |  |
| *pol2P287R* [pREP1-*Pfh1^+^* ] | EMM -thiamine | 1513 | 1193-1860 |
| *pol2P287R* [pREP1-*Pfh1^+^* ] | EMM +thiamine | 46 | 27-68 |
