# Supplementary material for: Expression of the cancer-associated DNA polymerase ε P286R in fission yeast leads to translesion synthesis polymerase dependent hypermutation and defective DNA replication
Source: PLoS Genet. 2021 Jul 6;17(7):e1009526. doi: 10.1371/journal.pgen.1009526 (PMC8284607; doi:10.1371/journal.pgen.1009526)
Supplement: Attachment — Submitted filename: responsereviewerscomments080621.docx [file pgen.1009526.s020.docx]

080621 v

**Soriano et al response to referees’ comments**

Comments to the Authors:
Please note here if the review is uploaded as an attachment.

Reviewer #1: The POLE P286R mutation is strongly associated with a hypermutation phenotype in human cancers. The precise reason why POLE P286R causes this phenotype is unknown. A number of previous studies have attempted to address this using various model systems include human, mouse and budding yeast, however, the question remains not fully resolved. The authors describe the use of a fission yeast model of the POLE P286R mutation. They show that the mutations generated in these mutants largely recapitulate human tumours (except the lack of mCpG in yeast). By testing the impact of different DNA damaging agents on the POLE mutant yeast, they conclude that these cells are highly sensitive to increase dNTP levels. Most significantly, they show that deletion of pol kappa, and to some extent pol eta, rescues the hypermutation phenotype, suggesting that the increased usage of these error-prone polymerases may be the reason why POLE mutants are hypermutated. Although these observations are not tested independently in other systems, nevertheless, it is an important contribution to our knowledge of POLE hypermutation. **I only have some relatively minor comments for the authors, which are listed below:**

1. The expected mutation rate should take in to account the GC content of the different regions. Ideally, something like SigProfilerSimulator (<https://bmcbioinformatics.biomedcentral.com/articles/10.1186/s12859-020-03772-3>) should be used to simulate the expected mutations and to account for trinucleotide content of the different regions.

1.1 The S. pombe genome is AT-rich, and lacks CpG islands and CpG methylation, so GC content is probably not a major determinant of mutagenicity. Nonetheless, the GC content is considered when we generate the normalized and humanized signatures for the subsequent analysis.

2. The comparison of % mutations in AT-rich regions should be more quantitative between pombe and humans. The % should be normalised by the size of the respective AT-rich regions. For instance, the fact that pombe has a higher proportion of the genome with >50bp rich should be accounted for.

1.2 Taking into account the reviewer’s comment, we find that in *POLE-P286R* human cancers, SNPs are enriched in AT-rich regions (>80%) <20 bp (S3 Fig, S6 Table). This is particularly significant in the case of TCT>TAT transversions. However, in S. pombe pol2-P287R, the distribution of the mutations in these regions is the same as expected based on the genome composition. We have deleted the original Fig 2D and the associated Fig S3, and modified the text (lines 204-208)

3. For the comparison of pombe and human P286R mutational signature, apart from using cosine similarity, it would be better to perform signature decomposition to get a more precise contribution of each signature in the pombe mutant.

1.3 The mutational signatures that contribute most to the pol2-P287R signature are SBS14 (26%), SBS20 (16%), SBS10a (11%) and SBS35 (11%). None of them are enriched in the wt. (S4 Table, text 223-224 )

4. “Cells with a high rate of spontaneous mutations frequently display increased sensitivity to DNA damaging agents [43, 44].” I don’t think this statement is necessarily true. For example, mismatch repair deficient cells (which have a high rate of spontaneous mutations) are resistant to temozolomide (<https://www.ncbi.nlm.nih.gov/pmc/articles/PMC3494444/>). The statement should be revised to perhaps specifically refer to DNA POLE mutants?

1.4 We have revised this sentence as follows: “Mutation of the N-terminus of Pol2 can lead to sensitivity to DNA damaging agents, possibly reflecting the role of Pol2 in various DNA repair pathways.” (lines 233-234)

5. When comparing the number of mutations with WT versus the kpa1 and eso1 mutants (Fig 7B), it is confusing to show the indels on the same plot because the reference mutation number of P287R alone is presumably just SNPs? It would be better for indels to be plotted separately. Furthermore, were there any replicates in these experiments? Error bars would be helpful.

1.5 These are now plotted separately and we have included error bars to show the range of data for the lineages analysed. We have indicated in the figure legend the number of replicates for each genotype.

Reviewer #2: In this manuscript, Soriano et al. present an analysis of the fission yeast DNA polymerase epsilon mutant P286R, orthologue of the corresponding POLE mutant identified as driver of hypermutated colorectal and endometrial human cancers. They show that these mutants show mutagenic phenotypes comparable to those of the corresponding human or S. cerevisiae pol epsilon mutants. Finally, they show that this mutant is hypersensitive to DNA damaging agents, that this sensitivity is modulated by the helicase PIF1 and that a large fraction of the mutagenic phenotype is due to the presence of trans-lesion DNA polymerase kappa.

The manuscript is largely well written, interesting, and the experiments have been appropriately designed. However, the bioinformatic analysis of mutation accumulation experiments has not been carried out to a sufficient depth, especially given that the mutations introduced by a strand-specific DNA polymerase are likely to present a positional bias associated with DNA replication origins (indeed it is the case for S. cerevisiae and mammalian cells). The most striking finding of this manuscript, the substantial suppression of the mutagenic phenotype of P286R cells by inactivation of pol kappa is relegated to the final figure and not well investigated or discussed.

I believe this manuscript could be improved by addressing the following points:

1. Page 4 line 101. The discussion of signatures SBS10a and SBS10b does not take into account that by now several authors in different organisms have shown that the signature produced by pol epsilon EDM mutants is SBS14 (Haradhvala et al., 2018; Tracy et al., 2020; Herzog et al.,2021). SBS10a/b, which are observed in some human cancers with pol epsilon EDM alleles (but not in the yeast counterparts) might instead originate from MMR-mediated skewing of SBS14 after it was introduced at the replisome of pol epsilon EDM cells.

2.1 We have cited these references (Herzog et al 2021 was published after we had completed the original version of our manuscript), and incorporated the reviewer’s suggestion (lines 101-103)

2. Page 5 line 112. Describing the “mutator effect” of pol2-P301R as “comparable with complete MMR deficiency” is potentially misleading, since the authors are clearly only referring to the mutation (accumulation?) rate and not to the mutational patterns. I suggest that the actual figures/rates are cited here.

2.2 We have changed the text to:

“and even in the heterozygous state the mutation rate to canavanine resistance is elevated 27 times relative to wildtype, comparable with complete MMR deficiency.”

3. Page 5 line 130-31. It has been shown that pol2-P301R does not lead to increased dNTP levels (Xing et al. 2019).

2.3 Thank you for pointing this out. We have deleted the sentence “However it remains unknown whether dNTP pool levels are elevated in response to cancer-associated Pol ε variants.”, and cited Xing et al. in the discussion of Fig 4B by including the sentence “Similarly, elevated dNTP levels were not found in the *S. cerevisiae pol2-P301R* strain”

4. Page 6 line 156-164. What does “on average” refer to (“On average, we identified 0.5, 9.5 and 394 single-base substitutions in…”)? Is it the average between different mutation accumulation lines of the same genotype? If so, please add standard deviation whiskers and/or plot every single MA line in fig 1C to show the dispersion around the average. What is the total number of variants studied in each genotype? Is it sufficient for the subsequent signature analyses?

Also, there seems to be a small increase in INDELS in P287R compared to the wild-type (a log scale, or separating INDELS and SBS in two different graphs would make it clearer). Is it statistically significant? It was shown recently that S. cerevisiae EDM mutants generate a large amount of insertions, which are almost completely masked by MMR, but can still be detected in pol2-P301R cells (Herzog et al, 2021).

2.4 See point 1.5. We now state in the figure legend the number of variant lineages analysed for each genotype. There is a statistically significant increase in the number of indels in P287R compared to wt.

5. Page 7 line 172. The authors should specify that this refers to the mutation spectrum of EDM alleles after it has been altered by MMR and possibly pol delta proofreading activity (Flood et al. 2015; Bulock et al. 2020).

2.5 We have changed the text to: “which is in accordance with the specific mutational spectrum of *POLE* EDMs, although this is after it has been altered by MMR and possibly Pol delta proofreading (lines 172-174).

6. Page 7 line 173-184: The authors should include a synthetic measure of the similarity of the different mutational patterns shown in fig 2A-B, such as cosine similarity after normalizing for the trinucleotide composition of each organism.

2.6 The cosine similarity between the mutational spectra of the P286R variant in human adenocarcinomas and S. pombe is 0.68. The pol2-P287R normalized and humanized signatures are almost identical, with a cosine coefficient of 0.98 (S4 Table). We used the humanized version for the comparison with the COSMIC signatures as previously described [1], but similar results would have been obtained using the normalized signatures.

7. Page 7 line 185-192. When discussing the nature of the mutational patterns observed in P287R cells, it should be noted that pol epsilon mutants only introduce mutations on the leading strand on which they operate. Therefore, for a meaningful discussion of the frequency of different mutational contexts, these should be reverse complemented when the corresponding mutations found on regions replicated as lagging strand (i.e. left of replication origins) to reflect their likely leading strand origin.

2.7 This analysis is not straightforward in S. pombe as, unlike the situation in S. cerevisiae, replication origin function is less deterministic, most origins showing a low probability of functioning in a given S phase, and comparing the same region of a chromosome, different sets of origins are used comparing one S phase to another [2]. Thus there is uncertainty whether a given template segment is replicated as a leading or lagging strand.

8. Page 8 lines 204-212. The rationale for this analysis is not well presented; and while this reviewer agrees with the general conclusion (“the mutagenic mechanism due to pol2-P287R is distinct from that seen in wild-type cells”), this conclusion does not seem to follow from the data presented in this paragraph and fig. 2D. It is also not well explained why the authors are focusing on the single TCT>TAT mutation and not on all the observed mutations, and it is not clear what one should conclude from the results presented.

2.8 Please see point 1.2, S Fig 3 and Table S6. The text has been amended as we cannot make the assertion that the mutagenic mechanism due to pol2-P287R is distinct from that seen in wild-type cells.

9. Page 8 line 227. The fact that that the mutational pattern of pol2-P287R has similarity with SBS14 even in the presence of functional MMR is not unexpected. This could reflect a differential efficiency of MMR-mediated alteration of SBS14 in fungi and mammalian cells. On a technical point, the low resolution of Figure 3B makes it difficult to judge the colour of adjacent signatures in this version of the pdf.

2.9 We have modified the text (lines 228-230) to accommodate the reviewer’s suggestion.

10. Page 9 line 251. See point 3. The equivalent mutation in S. cerevisiae has similarly been shown not to lead to increased dNTP levels (Xing et al. 2019).

2.10 See point 2.3.

11. Page10 Line 263. Given the similarity of the mutagenic mechanism of pol2-exo– and pol2-P287R, it would have been extremely interesting to show the mutational pattern in pol2-D276A/E278A cdc22-D57N (the only viable pol2 mutant) cells to determine the quantitative impact of increased dNTP levels on mutagenesis in different sequence contexts. The authors should at least show that this double mutant has an increased mutation rate compared to the single mutants by means of fluctuation tests.

2.11 Although pol2-P287R combined with cdc22-D57N is synthetically lethal, in extensive tetrad analysis we were able to pick a small pol2-P287R cdc22-D57N colony, presumably harbouring a suppressor mutation. When compared with the single pol2-P287R mutant, the double mutant shows high dNTP levels comparable to cdc22-D57N, an even higher mutation rate, and a growth defect on rich medium (S7 Table, S8 Table, and S4 Fig ).

12. Page 11 line 302. Does Pfh1 overexpression affect the mutation rate of pol2-P287R?

2.12 Pfh1 overexpression increases the mutation rate of pol2-P287R (S11 Table). This is in line with the observation that Pif1 overexpression increases the mutation rate in wt S. cerevisiae [3].

13. The substantial reduction in mutation rates of pol2-P287R observed with pol kappa mutants and to a lesser extent with pol eta mutants is extremely interesting, however several points could be addressed in more depth with experiments and/**or discussion**. For example:

a. Does pol2-P287R pol kappa pol eta triple mutant further reduce the mutation rate?

b. Is there any synthetic lethality in the triple mutant? If not, one should assume that other more accurate polymerases, perhaps pol delta, take over from stalled pol epsilon?

2.13ab Previous work has shown that mutation rates of S. pombe ddb1 and swi7 mutants, which are reduced by deleting either Pol kappa or zeta, are further reduced, by a small amount, by deleting both Pol kappa and zeta in a triple mutant [4, 5], so the prediction is that pol2P287R kpa1D eso1D would show a slight reduction in mutation rate compared to the double mutants.

c. If a large part of the mutations were indeed physically introduced by pol kappa in pol2-P287R cells, one would expect pol kappa to be responsible for “writing” SBS14, however SBS14 has been detected also in S. cerevisiae pol2-P301R MMR— strains, an organism that does not have a pol kappa homolog.

2.13c This is very good point. One possibility is that pol kappa (or some other TLS polymerase in S. cerevisiae) improves the efficiency of switching from stalled pol epsilon to pol delta on the leading strand. Pol delta synthesis on the leading strand is known to be mutagenic [6] and possibly could write SBS14. Interestingly, the next highest signature in terms of cosine similarity in both our work and that of Herzog et al is SBS20, known to be associated with pol delta mutations in the context of MMR inactivation [7]. Possibly MMR is not so efficient with DNA synthesised by pol delta on the leading strand, as old/new strand discrimination requires ribonucleotide incorporation, which is much lower with pol delta compared to pol epsilon [8]. We now discuss these points in the text (lines 368-395) and have modified the model (S6 Fig).

Reviewer #3: This MS from Steven Kearsey’s group examines the consequences of introducing the cancer prone POLε-P286R mutant into the fission yeast genome (the position is conserved as residue 287). This causes an increased mutation rate, and a mutation spectrum very similar to the one reported in human cancers. Interestingly, the mutations are suppressed by inactivation of translesion polymerases, suggesting that polymerase switching is involved. Consistent with this, it is shown by DNA combing and FACS analysis that replication proceeds slowly in the mutant, which is also synthetic lethal with absences of the Rad3 checkpoint. Finally, overexpression of the Pfh1 helicase can suppress some of the phenotypes associated with fission yeast P287R, whereas its deletion enhanced the phenotype.

Although I find this study interesting and compelling, I think there are some major problem with it in its present form. The work is manly confirmatory to previous studies in humans and budding yeast, but the potentially very interesting findings (see point 2 and 3 below) are not really pursued, despite the fact that fission yeast genetics should be most helpful in this process.

1. Although the mutation analysis is compelling, I missed a detailed discussion of the experimental setup chosen. Why was the cultures grown for 200 generations? What is the detection threshold for variant calling by the sequencing protocol? Were the identified mutants fixed in the population? Is phenotypic selection important in this time scale?

3.1 Choosing 200 generations was somewhat arbitrary, and a compromise between getting enough mutations to analyse, but avoiding acquisition of suppressor mutations in the pol2-P287R strain which could skew the mutational profile. Comparable studies have used 100-500 generation experiments [9, 10]. In MA experiment, cultures were bottlenecked to single cells between passages, so in the final sample, mutations would be expected to be fixed, i.e. in a high proportion of reads, except for mutations that were accumulated in the final culture used for DNA preparation. Variants were called if they were present in ≥ 20% reads.

2. In my opinion, the most interesting aspect of the paper is the reported co-lethality with the cdc22-D57N mutant defective in feedback inhibition of ribonucleotide reductase (RNR). The authors ascribe this to the combined mutation burden of the two mutagenic strain, but provide no evidence for this. However, cdc22-D57N can readily be combined with ddb1 and this gives rise to a strong mutagenic synergy; yet the double mutant is alive (Fleck et al 2013). Is the lethality suppressed by loss of TLS polymerases, which one would expect if the mutation burden were the problem? The co-lethality with cdc22-D57N should be pursued – for instance by a suppressor screen, or perhaps by testing if the D57N effect is dominant in a heterozygous context.

3.2 Please see point 2.11.
Also, to allow a direct comparison with the Fleck et al 2013 data, we have measured the ade6-485 reversion rate in the pol2-P287R background. This rate shows a 43±11 fold increase compared to wt, while the single ddb1D mutant shows a 25 fold increase [11], so this difference may account for error-induced extinction in pol2-P287R cdc22-D57N. Also the comparison between pol2-P287R and ddb1D is complicated by the fact that loss of Ddb1 leads to the stabilization the Spd1 inhibitor of RNR, so this will tend to reduce dNTP levels caused by cdc22-D57N. The suppression of synthetic lethality of pol2-P287R cdc22-D57N by deletion of genes for TLS polymerases is a possibility, given data in Fleck et al. (2013), but we have clarified the mechanism of pol2-P287R cdc22-D57N lethality (point 2.11). A suppressor screen would be very difficult as it would not be easy to distinguish suppressor mutations from the high background of passenger mutations in the hypermutagenic strain.

3. It is also very interesting that P287R is synthetically lethal with rad3, but I find it hard to believe that the observed weak activation of Cds1 and Chk1 can explain this. Again, a genetic analysis should be very informative.

3.3 We have included data showing that the pol2P287R rad3ts strain cuts when grown at 36°C (S5B Fig); presumably Rad3 is restraining mitotic entry, allowing completion of DNA repair/replication and consistent with partial Cds1 and Chk1 activation. We were able to derive rad3D pol2-P287R double mutants but these are synthetically sick compared to the single mutants (S5C Fig). We were also able to derive double cds1D pol2-P287R and chk1D pol2-P287R mutants (S5E Fig), but the double mutants were frequently slow growing, and in both these crosses and the rad3D cross it is possible that the double mutants are picking up suppressors that improve viability.

4. Why does the P287R strain have a much more severe phenotype than the strain lacking proofreading altogether? What is happening at the fork that causes checkpoint activation and polymerase switching in this mutant? ??

3.4 Please see point 2.13c and model presented in S6 Fig.

Minor comments:
L205: Delete “to see” - corrected
L245: an effect on -> effects This sentence has been redrafted

Have all data underlying the figures and results presented in the manuscript been provided?
Large-scale datasets should be made available via a public repository as described in the PLOS Genetics [data availability policy](http://journals.plos.org/plosgenetics/s/data-availability), and numerical data that underlies graphs or summary statistics should be provided in spreadsheet form as supporting information.

Reviewer #1: Yes

Reviewer #2: Yes

Reviewer #3: Yes

PLOS authors have the option to publish the peer review history of their article ([what does this mean?](https://journals.plos.org/plosgenetics/s/editorial-and-peer-review-process#loc-peer-review-history)). If published, this will include your full peer review and any attached files.


If you choose “no”, your identity will remain anonymous but your review may still be made public.


Do you want your identity to be public for this peer review? For information about this choice, including consent withdrawal, please see our [Privacy Policy](https://www.plos.org/privacy-policy).

Reviewer #1: Yes: Jason Wong

Reviewer #2: No

Reviewer #3: Yes: Olaf Nielsen

REFERENCES

1.	Meier B, Volkova NV, Hong Y, Schofield P, Campbell PJ, Gerstung M, et al. Mutational signatures of DNA mismatch repair deficiency in C. elegans and human cancers. Genome Research. 2018;28(5):666-75. doi: 10.1101/gr.226845.117.

2.	Kaykov A, Nurse P. The spatial and temporal organization of origin firing during the S-phase of fission yeast. Genome Res. 2015;25(3):391-401. Epub 2015/02/05. doi: 10.1101/gr.180372.114. PubMed PMID: 25650245; PubMed Central PMCID: PMCPMC4352884.

3.	Ahn S-M, Adnan Ahmad A, Kim J, Kim D, Chun S-M, Kim J, et al. The somatic POLE P286R mutation defines a unique subclass of colorectal cancer featuring hypermutation, representing a potential genomic biomarker for immunotherapy. Oncotarget. 2016;7(42):68638-49. doi: 10.18632/oncotarget.11862.

4.	Kai M, Wang TSF. Checkpoint activation regulates mutagenic translesion synthesis. Genes & development. 2003;17(1):64-76. doi: 10.1101/gad.1043203.

5.	Holmberg C, Fleck O, Hansen HA, Liu C, Slaaby R, Carr AM, et al. Ddb1 controls genome stability and meiosis in fission yeast. Genes Dev. 2005;19(7):853-62. Epub 2005/04/05. doi: 10.1101/gad.329905. PubMed PMID: 15805471; PubMed Central PMCID: PMCPMC1074322.

6.	Garbacz MA, Cox PB, Sharma S, Lujan SA, Chabes A, Kunkel TA. The absence of the catalytic domains of Saccharomyces cerevisiae DNA polymerase strongly reduces DNA replication fidelity. Nucleic Acids Res. 2019;47(8):3986-95. Epub 2019/01/31. doi: 10.1093/nar/gkz048. PubMed PMID: 30698744; PubMed Central PMCID: PMCPMC6486559.

7.	Haradhvala NJ, Kim J, Maruvka YE, Polak P, Rosebrock D, Livitz D, et al. Distinct mutational signatures characterize concurrent loss of polymerase proofreading and mismatch repair. Nat Commun. 2018;9(1):1746. Epub 2018/05/03. doi: 10.1038/s41467-018-04002-4. PubMed PMID: 29717118; PubMed Central PMCID: PMCPMC5931517.

8.	Lujan SA, Williams JS, Clausen AR, Clark AB, Kunkel TA. Ribonucleotides are signals for mismatch repair of leading-strand replication errors. Mol Cell. 2013;50(3):437-43. Epub 2013/04/23. doi: 10.1016/j.molcel.2013.03.017. PubMed PMID: 23603118; PubMed Central PMCID: PMCPMC3658170.

9.	Herzog M, Alonso-Perez E, Salguero I, Warringer J, Adams DJ, Jackson SP, et al. Mutagenic mechanisms of cancer-associated DNA polymerase alleles. Nucleic Acids Res. 2021;49(7):3919-31. Epub 2021/03/26. doi: 10.1093/nar/gkab160. PubMed PMID: 33764464; PubMed Central PMCID: PMCPMC8053093.

10.	Tracy MA, Lee MB, Hearn BL, Dowsett IT, Thurber LC, Loo J, et al. Spontaneous Polyploids and Antimutators Compete During the Evolution of Saccharomyces cerevisiae Mutator Cells. Genetics. 2020;215(4):959-74. Epub 2020/06/10. doi: 10.1534/genetics.120.303333. PubMed PMID: 32513814; PubMed Central PMCID: PMCPMC7404223.

11.	Fleck O, Vejrup-Hansen R, Watson A, Carr AM, Nielsen O, Holmberg C. Spd1 accumulation causes genome instability independently of ribonucleotide reductase activity but functions to protect the genome when deoxynucleotide pools are elevated. Journal of cell science. 2013;126(Pt 21):4985-94. doi: 10.1242/jcs.132837.
